# The Effects of Polymer–Nitrogen Fertilizer on Biomes in Drip-Irrigated Wheat Soil

**DOI:** 10.3390/microorganisms13061334

**Published:** 2025-06-09

**Authors:** Yan Sun, Chunying Wei, Shenglin Zhang, Hua Fan, Dashuang Hong, Hong Huang, Kaiyong Wang

**Affiliations:** 1 Agricultural College, Shihezi University, Shihezi 832000, China; sunyan72@xjshzu.cn.com (Y.S.); weichunying@xjshzu.com (C.W.); fanhua@shzu.edu.cn (H.F.); 20192012047@stu.shzu.edu.cn (D.H.); 2 State Key Laboratory of Efficient Utilization for Low Grade Phosphate Rock and Its Associated Resources, Guiyang 550000, China; zhangshenglin1978@outlook.com

**Keywords:** polymer, wheat, nematode diversity, nitrogen conversion, nitrogen reduction

## Abstract

Polymer application combined with nitrogen (N) fertilization can increase soil N transformation efficiency. However, the mechanism of polymer influencing soil biocommunity characteristics and nitrogen transformation is still unclear. In this field experiment, a self-developed water-soluble polymer material (PPM, a mixture of anionic polyacrylamide, polyvinyl alcohol, and manganese sulfate) was combined with N fertilization N100 (300 kg/hm^2^ of N), PN100 (PPM + 300 kg/hm^2^ of N), and PN80 (PPM + 240 kg/hm^2^ of N) to investigate soil biodiversity, enzyme activities, and metabolomics. The results showed that under the application of PPM, the contents of soil total nitrogen (TN), alkali hydrolyzable nitrogen (ANS), nitrate nitrogen, organic carbon (SOC), and microbial biomass nitrogen (MBN) increased with a decrease in the N application rate, while soil bulk density, pH, and EC (electrical conductivity) decreased. The Chao 1 index of soil bacterial and nematode communities of the PN80 treatment was 30.6% and 10.7% higher than that of the N100 treatment, respectively, and the Shannon index was 2.72% and 2.64% higher than that of the N100 treatment, respectively. In the short term, the application of PPM affected the structure and composition of soil bacterial and nematode communities. In particular, the relative abundances of omnivorous (*Aporcelaimellus*) and bacterivorous (*Prismatolaimus*) nematodes were significantly higher than those of the N100 treatment. These changes further regulated the soil metabolites, promoting soil nitrogen transformation. This study will provide a scientific basis for nitrogen reduction in drip-irrigated wheat planting in arid regions.

## 1. Introduction

Wheat is one of the most important food crops in the world. Many studies have shown that wheat root-zone biocommunities are closely related to wheat growth and health [1]. Soil microorganisms and nematodes are important components of soil biocommunities and play a vital role in ecosystem functions [2]. Long-term application of inorganic fertilizers significantly affects the structure of soil bacterial and fungal communities and inhibits the biomass accumulation of nematodes (especially fungivorous nematodes) [3]. For example, the application of both chemical and organic fertilizers increased soil nutrients and bacterial diversity compared with the application of chemical fertilizer [4,5]. Long-term application of organic fertilizers could maintain the bacterial diversity in low-productivity soils, increasing the availability of nutrients in the soil. Soil quality is significantly affected by nitrogen fertilization. Studies have shown that long-term nitrogen fertilization could significantly increase soil microbial biomass and promote the growth of soil fungi compared with the unfertilized control group [6]. However, excessive nitrogen fertilization could acidify the soil, interfere with soil enzyme activity and nutrient cycling, and indirectly affect soil microbial communities [7]. Moreover, soil acidification may further lead to a reduction in the abundance and diversity of soil microorganisms [8]. Therefore, it is of great significance to study the effects of chemical nitrogen-use efficiency on wheat growth and soil biocommunity.

The fertilizer-use rate is very low due to inappropriate fertilization, especially excessive nitrogen application, causing serious damage to the ecosystem [9]. To meet the needs of crop growth and increase crop yield, it is necessary to optimize nitrogen fertilization [10]. However, due to their low thermal stability and high solubility [11], nitrogen fertilizers are rapidly hydrolyzed after direct application to the soil, which affects the soil nitrogen availability in the later growth stage of crops. Polymers have been used to regulate soil moisture in recent years. Polymers can improve the soil moisture status to regulate the transport of solutes in the soil, and can also adsorb nutrients in the soil and fertilizers. The combined application of polymers and nitrogen fertilizer can increase the availability of nitrogen [12].

Soil microbial diversity is associated with soil quality and greatly impacts crop productivity [13]. Previous studies have shown that polymer application can increase soil microbial diversity by promoting the formation of large soil aggregates, increasing soil water content, pH, EC, bulk density, and porosity, and inhibiting nutrient loss compared with the control [14,15]. Polymers can increase the relative abundances of *Acidobacteria*, *Gemmatimonadetes*, and *Nitrospira* compared with the control [14]. Therefore, polymers have a good function of improving the soil microenvironment. In addition, many studies have shown that long-term excessive nitrogen application can stimulate many metabolic processes in the soil, especially carbohydrate and amino acid metabolism [16]. Nitrogen fertilization in a certain amount can increase the abundance of genes related to carbohydrate metabolism compared with the control, but excessive nitrogen fertilization can increase the abundance of nitrate reductase activity genes in microbial communities [17]. The mechanism of polymer application impacting soil physicochemical properties and microbial communities is not yet clear.

At present, fertilization through drip irrigation systems is very popular in arid areas such as Xinjiang, China. However, most polymers are solid and difficult to apply through drip irrigation systems [18]. This leads to a difficulty for polymers to fully contact with fertilizers, affecting their application performance [19]. Fortunately, a liquid-modified polymer with water-retaining and slow-release functions was successfully developed by us (PPM, patent number: CN105801297, composed of polyacrylamide, polyvinyl alcohol, and manganese sulfate). Previous studies have explored the effects of nitrogen-fertilizer-use efficiency on crop growth and soil microorganisms, as well as the slow-release effect of polymers on soil nutrients. However, there is currently no in-depth analysis of the effects of polymers, especially PPM (a new water-soluble polymer), on crop nitrogen utilization and soil biocommunity. Therefore, in this study, the new polymer PPM was combined with different nitrogen fertilization rates. The specific objectives were to clarify the effects of the combined application of PPM and nitrogen fertilizer on soil physicochemical properties, enzyme activities, microbial and nematode diversity and abundance, and carbon and nitrogen metabolism characteristics. This study will provide a technical reference for nitrogen reduction and nitrogen-use efficiency enhancement in drip-irrigated wheat fields in arid regions.

## 2. Materials and Methods

### 2.1. Experimental Site

The experiment was conducted from March to July in 2021 at the experimental field of Shihezi University, Xinjiang, China (44°33′ E, 86°05′ N). This region has a temperate continental climate. The annual sunshine hours were 2721–2818 h. The average annual temperature was 2–15 °C. The average annual rainfall and evaporation were 180–220 mm and 1000–1500 mm, respectively. The soil type was Calcaric Fluvisals, with a texture of loam [20]. The pH (soil:water = 1:2.5) was 7.51, the electrical conductivity (EC) was 344 µS/cm, the total N content was 1.76 g/kg, the alkali hydrolyzable N content was 70.2 mg/kg, the available phosphorus content was 18.2 mg/kg, and the available potassium content was 204 mg/kg [12].

### 2.2. Experimental Design

The independently developed polymer, PPM (TRent No: CN105801297), is synthesized by polyacrylamide, polyvinyl alcohol, and manganese sulfate. It has water-retention and sustained-release properties, with a pH of 7.46, and an EC of 1330 µS/cm. Polyacrylamide (PAM, C_3x_H_5x_N_x_O_x_), a linear anionic polymer with a molecular weight of 8–10 million, was purchased from McLean Biochemical Technology Co., Ltd. (Chongqing, China). Polyvinyl alcohol, MnSO_4_, H_2_SO_4_, and H_2_O_2_ were analytically pure (AR) and purchased from Jinbei Fine Chemicals Co., Ltd. (Tianjin, China) [12]. The spring wheat cultivar XC38 is a high-yield cultivar with strong gluten characteristics jointly developed by the Crop Research Institute of Xinjiang Academy of Agricultural Reclamation Science and China Shihezi Jiuhe Seed Industry Co., Ltd. (Shihezi, China), with a growth period of 94 days [21].

There were three treatments in the experiment, and a randomized complete block design was employed (Table 1). Each treatment had three replicates. The area of each plot was 10 m^2^ (2 m × 5 m), and the plot spacing was 0.5 m. Wheat seeds were sown on 30 March 2021, and 120 kg/hm^2^ of phosphate fertilizer (NH_4_H_2_PO_4_, P_2_O_5_: 48%, Guizhou Phosphor (Group) Co., Ltd. (Guiyang, China)) and 90 kg/hm^2^ of potash fertilizer (K_2_SO_4_, K_2_O: 51%) were applied before sowing through a drip irrigation system. PPM was dissolved in irrigation water in a fertilizer tank with N fertilizer (Urea: 46%) (Table 1), and applied to the soil after wheat seedling emergence. The total irrigation amount in the whole growth period of wheat was 4500 m^3^·hm^2^. Other agricultural managements were consistent with those in local fields. Wheat was harvested on 7 July 2021.

### 2.3. Soil Sampling

Soil samples (0–20 cm soil layer) were collected during the flowering stage of wheat (8–16 June). Three soil samples were collected from each plot using a soil drill. After mixing the three soil samples, air-drying, and sieving (2 mm), the soil sample of each plot was divided into two parts. Part 1 was stored at 4 °C for the determination of soil physicochemical properties and enzyme activity. Some soil samples of Part 1 were stored in aluminum boxes for soil bulk density (BD) analysis, while the remaining soil samples were stored in plastic containers (Xinxiang Jingjin Company, Xinxiang, China), filtered through an 8 mm sieve, and air-dried for soil aggregate analysis. Part 2 was stored at −80 °C for analysis of soil microbiomes and metabolome.

### 2.4. Measurements

#### 2.4.1. High-Throughput Sequencing

Soil high-throughput sequencing was conducted to determine soil microbial and nematode composition and abundance. Total genomic DNA extraction was conducted using the FastDNA^®^Spin Kit for sediment (MP Biomedicals, Santa Ana, CA, USA). For the analysis of soil bacterial diversity, the V3–V4 region of the 16S rRNA gene (forward primer: 5′-ACTCCTACGGGGAGGCAGCA-3′; reverse primer: 5′-GGACTACHVGGGTWTCTAAT-3′) was selected for PCR amplification. For the analysis of fungal diversity, ITS1 (forward primer: 5′-GGAAGTAAAAGTCGTAACAAGG-3′; reverse primer: 5′-GCTGCGTTCTTCATCGATGC-3′) was selected for PCR amplification. For the analysis of nematode diversity, the ITS region of the 18S rRNA gene (forward primer: 5′-GGGTGGTGCATGGGCCGTTCTTAGTT-3′, reverse primer: 5′-GCTGCGTTCTTCATCGATGC-3′) was selected for PCR amplification. The concentration and purity of the extracted DNA were determined using Qubit (Thermo Fisher Scientific, Waltham, MA, USA) and Nanodrop (Thermo Fisher Scientific, Weihao, Chicago, IL, USA) instruments. Genome sequencing was conducted by Personal Biotechnology Company (Shanghai, China) using the Pacific Biosciences platform and the Illumina Novaseq platform.

#### 2.4.2. Metabolome Measurement

Non-targeted metabolomics analysis was conducted using ultra-high performance liquid chromatography–mass spectrometry (UPLC-MS). Soil samples (200 mg) were vortexed and shaken for 30 s in a 2 mL EP tube with 0.6 mL of 2-chlorophenylalanine (4 ppm)-methanol (−20 °C) solution. Subsequently, 100 mg of glass beads was added, placed in the CBGT-48 high-throughput tissue grinder, and ground at 60 Hz for 90 s. Samples were sonicated for 30 min at room temperature and placed on ice for 30 min to allow complete precipitation of metabolites. The samples were centrifuged at 12,000 rpm at 4 °C for 10 min, and 300 uL of supernatant was collected and passed through a 0.22 μm filter. To monitor system stability and correct system bias, 20 μL of each sample was taken and mixed for QC (quality control) sample preparation. Chromatographic separations were accomplished on an Ultimate 3000 (Thermo, UDA, Waltham, MA, USA) system with an ACQUITY UPLC^®^HSS T3 column (150 mm × 2.1 mm, 1.8 µm, Waters Corporation, Milford, MA, USA). Mass spectrometry was performed using a mass spectrometer (Q Exactive HF-X, Thermo, USA) in positive and negative modes [22]. Peak identification, peak filtering, and peak alignment were performed on the raw data using the XCMS package in R software (version 3.6.1, Boston, MA, USA) (https://www.r-project.org/, accessed on 28 August 2021), to obtain the data matrix x, including mass-to-charge ratio (*m*/*z*) and retention time (rt). Batch normalization of peak areas was performed to compare data of different magnitudes.

#### 2.4.3. Measurement of Soil Enzyme Activity

Soil catalase activity was determined by KMnO_4_ titration [23]. Soil urease activity was determined by indophenol blue-colorimetry at 578 nm [24]. Soil protease activity was determined by the ninhydrin colorimetric assay [25]. Soil nitrate reductase activity was determined by phenol disulfonic acid-colorimetry at 520 nm [26]. Soil nitrite reductase activity was determined by α-namine-colorimetriy at 520 nm [27]. Soil hydroxylamine reductase activity was determined by the ferric ammonium sulfate-phenthroline colorimetry at 510 nm [24].

#### 2.4.4. Measurement of Soil Microbial Biomass Carbon and Nitrogen

Soil microbial biomass carbon (MBC) and nitrogen (MBN) were measured by the chloroform fumigation–extraction method [28]. Two fresh soil samples (12.5 g) of each treatment were placed in two plastic bottles for fumigation and non-fumigation treatment, respectively. The plastic bottles were placed in a vacuum dryer, with one bottle containing chloroform (with a small amount of glass beads in the beaker), the other bottle containing water, and a cup of diluted NaOH solution. Then, a vacuum device was used for vacuuming, making chloroform boil vigorously for 3–5 min. After closing the valve of the vacuum dryer, fumigation was conducted under dark conditions at 25 °C for 24 h. The soil samples of the non-fumigation treatment were placed in another dryer. After fumigation, the samples were extracted with 0.5 mol/L K_2_SO_4_ solution, shaken, filtered, followed by the determination of the total soluble C and N in the extract using a Multi C/N analyzer (Multi C/N 3000, Analytik, Jena, Germany) [29].

#### 2.4.5. Measurement of Soil Aggregates

After bringing the soil samples back to the laboratory, small stones and animal and plant residues were removed from the samples, and the samples were divided into small blocks of different sizes along the natural profile. The soil blocks were placed in a ventilated place for air drying. Then, 150 g of air-dried soil samples was weighed and sieved through 5.0, 2.0, 1.0, 0.5, 0.25, and 0.053 mm sieves. After that, the samples were oscillated on an oscillator at 180 r/min for 5–10 min to obtain soil aggregates of different particle sizes. This step was repeated five times [30].

The content of soil aggregates was calculated by the following equation [31]:

(1) $$SAC=\frac{MA}{TA}\times 100\%$$

where SAC (soil aggregate content) represents the mass percentage of aggregates of each particle size, MA (mass of aggregate) represents the mass of aggregates of a certain particle size, and TA (total aggregate) represents the total mass of aggregates [32].

The stability of soil aggregates was characterized by mean weight diameter (MWD, mm) and geometric mean diameter (GMD, mm):

(2) $$R_{0.25}=\sum_{n=1}^{i}\left(Wn\right)$$

(3) $$MWD=\sum_{n=1}^{i}\left(\bar{X}\times Wn\right)$$

(4) $$GMD=exp\left(\sum_{n=1}^{i}Ln\bar{X}\times Wn\right)$$

where R_0.25_ represents the content of aggregates with a diameter greater than 0.25 mm, $\bar{X}$ is the average diameter (mm) of soil aggregates within a certain particle size range, and Wn is the mass percentage of soil aggregates of size *n*.

#### 2.4.6. Soil Physicochemical Properties

The physicochemical properties of soil were determined according to the methods proposed by Bao [33]. Soil organic carbon (SOC) content was determined by external heating with potassium dichromate and concentrated sulfuric acid. Soil pH was determined by using a pH meter (Leimage PHS-3C, Shanghai, China) with a water–soil volume ratio of 5:1. Soil electrical conductivity (EC) was determined by using a conductivity meter (MP521, Sanxin, Shanghai, China) with a water–soil volume ratio of 5:1. Soil bulk density (BD) was determined by the cutting-ring method. Soil total nitrogen (TN) content was determined by the concentrated H_2_SO_4_ digestion Kjeldahl method using a Kjeldahl meter (Foss KjeltecTM2300, Hoganas, Sweden) [34]. Soil ammonium nitrogen (NH_4_^+^–N) content was determined by indophenol blue-based colorimetry–spectrophotometer (UV9000, Metash, Shanghai, China, absorbance value was determined at 420 nm). Soil nitrate nitrogen (NO_3_^−^–N) content was determined using dual-wavelength UV spectrophotometry (220 nm/275 nm). Soil available potassium (AK) content was determined using ammonium acetate extraction-flame photometry (FP6440, Yoke Instruments, Shanghai, China). Soil available phosphorus (AP) content was determined using sodium bicarbonate extraction–molybdenum antimony colorimetry (UV9000 spectrophotometer). Soil alkali hydrolyzable nitrogen (AN) content was determined using the alkaline diffusion method.

### 2.5. Data Analysis

All data were statistically analyzed in SPSS software (version 22.0). One-way analysis of variance (ANOVA) was conducted to determine the significance of the differences across the treatments (*p* < 0.05), and Duncan’s multiple comparison test was conducted to identify specific groups with differences (*p* < 0.05). Redundancy analysis (RDA) was conducted using the “vegan” package in R software (version 3.6.1) to reveal the relationship between soil bacterial, fungal, and nematode community structure and soil physicochemical properties. Linear discriminant analysis effect size (LDA Effect Size) was used to select microbial communities with significant differences between treatments, with the LDA threshold being 2.4. The criteria VIP (variable importance in projection) > 1 and 0.05 < *p* < 1 (single-factor analysis) were used for selecting soil metabolites with significant differences in abundance. All detected soil metabolites were functionally annotated using the Kyoto Encyclopedia of Genes and Genomes (KEGG) database (https://www.kegg.jp/, accessed on 15 October 2021). Spearman correlation coefficients were calculated using “psych” in R software (version 3.6.1) to further analyze the correlation between soil microbial communities and metabolites, and the correlations with a coefficient (Pearson) greater than 0.9 were retained. The co-occurrence networks were visualized through Gephi software (version 0.9.2) (https://gephi.org/, accessed on 2 November 2021). Data sorting was completed in Excel 2019, and chart drawing was completed using R software (version 3.6.1) and OriginPro 2018 software.

## 3. Results

### 3.1. Effects of Combined Application of PPM and Nitrogen Fertilizer on the α-Diversity Indices of Soil Biocommunity

**Figure 1:**
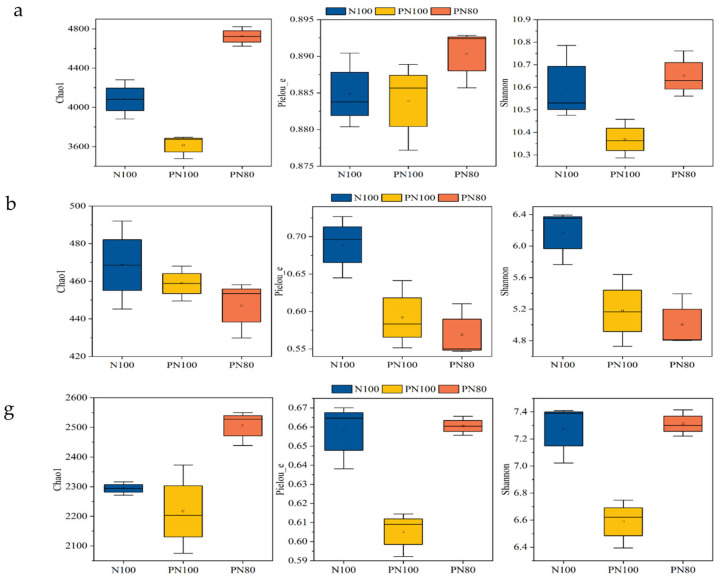

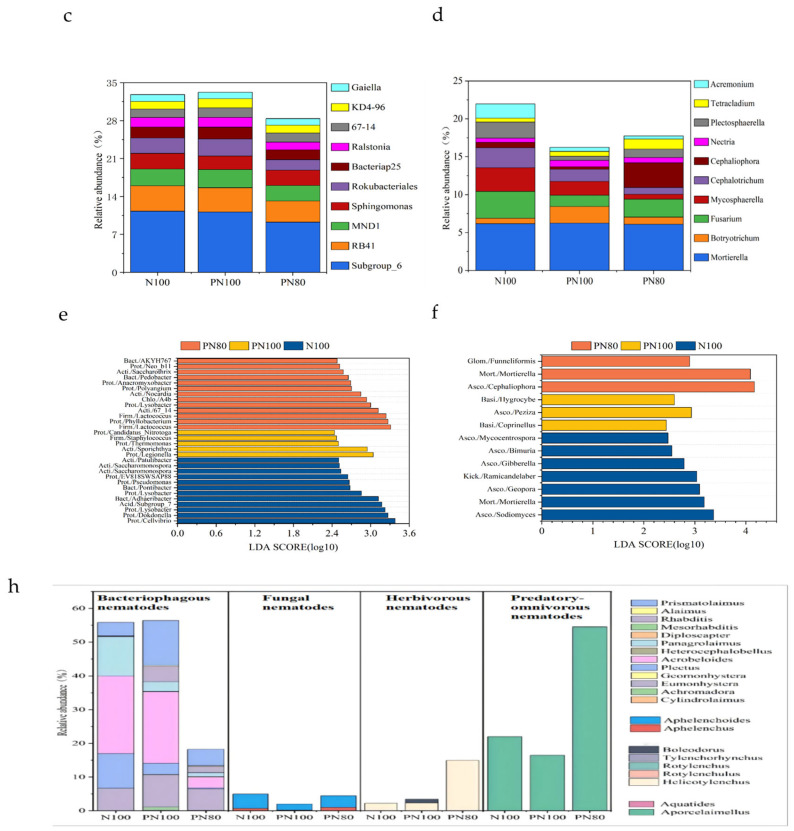
Soil microbial community richness and diversity under combined application of polymer PPM and nitrogen fertilizer: (**a**) Soil bacterial diversity index under different treatments; (**b**) Soil fungal diversity index under different treatments; (**c**) Soil bacterial community structure and abundance under different treatments; (**d**) Soil fungal community structure and abundance under different treatments; (**e**) LefSe analysis of soil bacteria in different treatments; (**f**) LefSe analysis of soil fungi in different treatments; (**g**) Changes in soil nematode diversity index under different treatments; (**h**) Changes in soil nematode community composition and abundance under different treatments.

The application of PPM had different effects on the Chao 1 index of soil biocommunities of different treatments (*p* < 0.05) (Figure 1a). The Chao 1 index of soil bacterial and nematode communities of the PN80 treatment was 30.6% and 10.7% greater than that of the N100 treatment, respectively (*p* < 0.05) (Figure 1a,g). However, there was no difference in the Chao 1 index of soil fungal community between the treatments (*p* > 0.05) (Figure 1b). The application of PPM also had different effects on the Shannon 1 index of soil bacterial and nematode communities of different treatments. The Shannon index of soil bacterial and nematode communities of the PN80 treatment was 2.72% and 3.17% greater than that of the N100 treatment, respectively (*p* < 0.05) (Figure 1a,g). The combined application of PPM and nitrogen fertilizer significantly impacted the Pielou and Shannon indexes. The Pielou and Shannon indexes of the PN100 treatment were 21.1% and 23.3% smaller than those of the N100 treatment, respectively (*p* < 0.05), and the Pielou and Shannon indexes of the PN80 treatment were 17.4% and 18.9% smaller than those of the N100 treatment, respectively (*p* < 0.05) (Figure 1b).

### 3.2. Effects of Combined Application of PPM and Nitrogen Fertilizer on the Soil Biocommunity Structure and Abundance

The dominant soil bacterial genera of the three treatments were *Subgroup_6*, *RB41* (*Acidobacteria*), *MND1*, *Sphingomonas*, *Bacteriap25*, *Ralstonia* (*Proteobacteria*), *Rokubacteriales* (*Rokubacteria*), *KD4-96* (*Chloroflexi*), *67-14*, and *Gaiella* (*Actinobacteria*) (Figure 1c,d). The relative abundances of soil bacteria *Subgroup_6*, *Rokubacteria*, and *Ralstonia* (*Proteobacteria*) of the PN100 treatment were 17.9%, 32.1%, and 18.8% lower than those of the N100 treatment, respectively (*p* < 0.05), but the relative abundance of *KD4-96* (*Chloroflexi*) of the PN80 treatment was 19.7% higher than that of the N100 treatment (*p* < 0.05).

The dominant soil fungal genera of the three treatments were *Mortierella* (*Mortierellomycota*), *Botryotrichum*, *Fusarium*, *Mycosphaerella*, *Cephalotrichum*, *Cephaliophora*, *Nectria*, *Plectosphaerella*, *Tetracladium* (*Ascomycota*), and *Acremonium* (Figure 1d). The relative abundance of *Fusarium* of the PN100 and PN80 treatments was 57.9% and 33.4% lower than that of the N100 treatment, respectively (*p* < 0.05). Similarly, the relative abundances of *Cephalotrichum*, *Plectosphaerella*, and *Acremonium* of the PN100 treatment were 39.2%, 74.3%, and 70.7% lower than those of the N100 treatment, respectively (*p* < 0.05), and the relative abundances of *Cephalotrichum*, *Plectosphaerella*, and *Acremonium* of the PN80 treatment were 66.3%, 47.6%, and 77.8% lower than those of the N100 treatment, respectively (*p* < 0.05). In addition, the relative abundances of *Botryotrichum* and *Nectria* of the PN100 treatment were 207% and 50.1% higher than those of the N100 treatment, respectively (*p* < 0.05). The relative abundance of *Mycosphaerella* of the PN80 treatment was 79.2% lower than that of the N100 treatment (*p* < 0.05), and the relative abundance of *Cephaliophora* was 365% higher than that of the N100 treatment (*p* < 0.05).

A total of 22 nematode genera were identified in the soils of the three treatments, including 13 bacteriophagous nematodes, 2 fungivorous nematodes, 5 herbivorous nematodes, and 2 predatory/omnivorous nematodes. The relative abundances of the fungivorous nematode *Aphelenchoides* and the predatory/omnivorous nematode *Aporcelaimellus* of the PN100 treatment were 153% and 34.1% lower than those of the N100 treatment, respectively (*p* < 0.05), and the relative abundance of the fungivorous nematode *Prismatolamius* was 229% higher than that of the N100 treatment (*p* < 0.05). Surprisingly, the relative abundances of the herbivorous nematode *Helicotylenchus* and the predatory/omnivorous nematode *Aporcelaimellus* of the PN80 treatment were 577% and 147% higher than those of the N100 treatment, respectively (*p* < 0.05). Moreover, bacteriophagous nematodes *Alamius*, *Geomonhystera*, and *Achromadora* and the herbivorous nematode *Boleodorus* were only detected in the PPM treatments.

### 3.3. Analysis of Differential Abundance of Soil Bacterial and Fungal Communities

LEfSe differential analysis (LDA Effect Size) was conducted to further compare the key bacteria that have significant roles in different treatments. The PN80 treatment had the most complex microbial community changes. A total of 30 key bacterial genera were identified in the three treatments (Figure 1e). LEfSe analysis found that the PN100 and PN80 treatments had more soil bacteria and fungi than the N100 treatment (*p* < 0.05). In the N100 treatment, *Cellvibrio*, *Dokdonella*, *Lysobacter*, *Pseudomonas*, *EV818SWSAP88* (*Proteobacteria*), *Adhaeribacter*, *Pontibacter* (*Bacteroidetes*), *Saccharomonospora*, *Patulibacter* (*Actinobacteria*), and *Subgroup_7* (*Acidobacteria*) had a larger LDA value compared with other bacterial genera. In the PN100 treatment, *Legionella*, *Thermomonas*, *Candidatus_Nitrotoga* (*Proteobacteria*), *Staphylococcus* (*Firmicutes*), and *Sporichthya* (*Actinobacteria*) had a larger LDA value. In the PN80 treatment, *Phyllobacterium*, *Lysobacter*, *Polyangium*, *Anaeromyxobacter*, *Neo_b11* (*Proteobacteria*), *Lactococcus* (*Firmicutes*), *A4b* (*Chloroflexi*), *Pedobacter*, *AKYH767* (*Bacteroidetes*), *67_14*, *Nocardia*, and *Saccharothrix* (*Actinobacteria*) had a larger LDA value. To determine the key fungi significantly affected by the treatments, LefSe analysis was further conducted, revealing a total of 13 key fungal genera in the treatments (Figure 1f). In the N100 treatment, *Sodiomyces*, *Geopora*, *Gibberella*, *Bimuria*, *Mycocentrospora* (*Ascomycota*), *Coprinellus* (*Basidiomycota*), *Ramicandelaber* (*Kickxellomycota*), and *Mortierella* (*Mortierellomycota*) had a larger LDA value compared with other fungal genera. In the PN100 treatment, *Peziza* (*Ascomycota*) and *Hygrocybe* (*Basidiomycota*) had a larger LDA value. In the PN80 treatment, *Cephaliophora* (*Ascomycota*), *Funneliformis* (*Glomeromycota*), and *Mortierella* (*Mortierellomycota*) had a larger LDA value. Therefore, the soil microbial community structures of the PN100 and PN80 treatments were generally similar, but there were some differences in the relative abundance of the microbial communities (Figure 1f).

### 3.4. Effects of Combined Application of Polymer and Nitrogen Fertilizer on Soil Physicochemical Properties and Enzyme Activities

**Figure 2:**
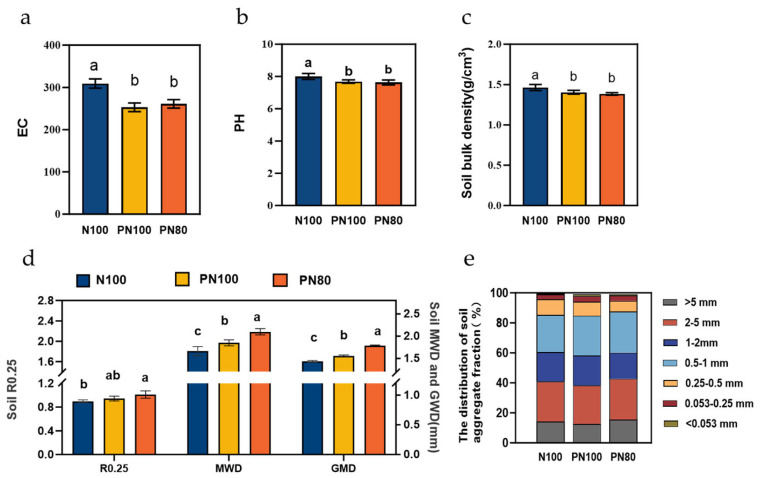
Changes in soil EC (**a**), pH (**b**), bulk density (**c**), aggregate stability (**d**), and aggregate particle size distribution (**e**) under different treatments. R0.25, soil aggregates with a particle size greater than 0.25 mm; GMD, geometric mean diameter; MWD, mean weight diameter. Different lowercase letters indicate significant difference between groups (*p* < 0.05).

The application of PPM significantly affected the particle size distribution of soil aggregates (Figure 2e) and significantly reduced soil bulk density (Figure 2c). The contents of soil aggregates with a particle size small than 0.053 mm and in the ranges of 0.053–0.25 mm and 0.5–1.0 mm of the PN80 and PN100 treatments were 35.9–76.5%, 12.2–14.0%, and 6.93%–11.4% higher than those of the N100 treatment, respectively (*p* < 0.05). In addition, the content of soil aggregates with a particle size greater than 0.25 mm (R0.25), geometric mean diameter (GMD), and mean weight diameter (MWD) of the PN80 treatment were 5.10%, 6.78%, and 8.91% higher than those of the N100 treatment, respectively, and the R0.25, GMD, and MWD of the PN100 treatment were 7.15%, 11.4%, and 11.1% higher than those of the N100 treatment, respectively (*p* < 0.05, Figure 2d). In addition, the soil bulk density of the PN100 and PN80 treatments was 0.06 and 0.02 g·cm^−3^ lower than that of the N100 treatment (*p* < 0.05, Figure 2c). The application of PPM had significant effects on soil physicochemical properties, i.e., soil EC and pH. The soil pH of the PN100 and PN80 treatments was 0.31 and 0.36 lower than that of the N100 treatment, respectively (*p* < 0.05, Figure 2a), and the soil EC was 18.2% and 15.5% lower than that of the N100 treatment, respectively (*p* < 0.05, Figure 2b).

**Figure 3:**
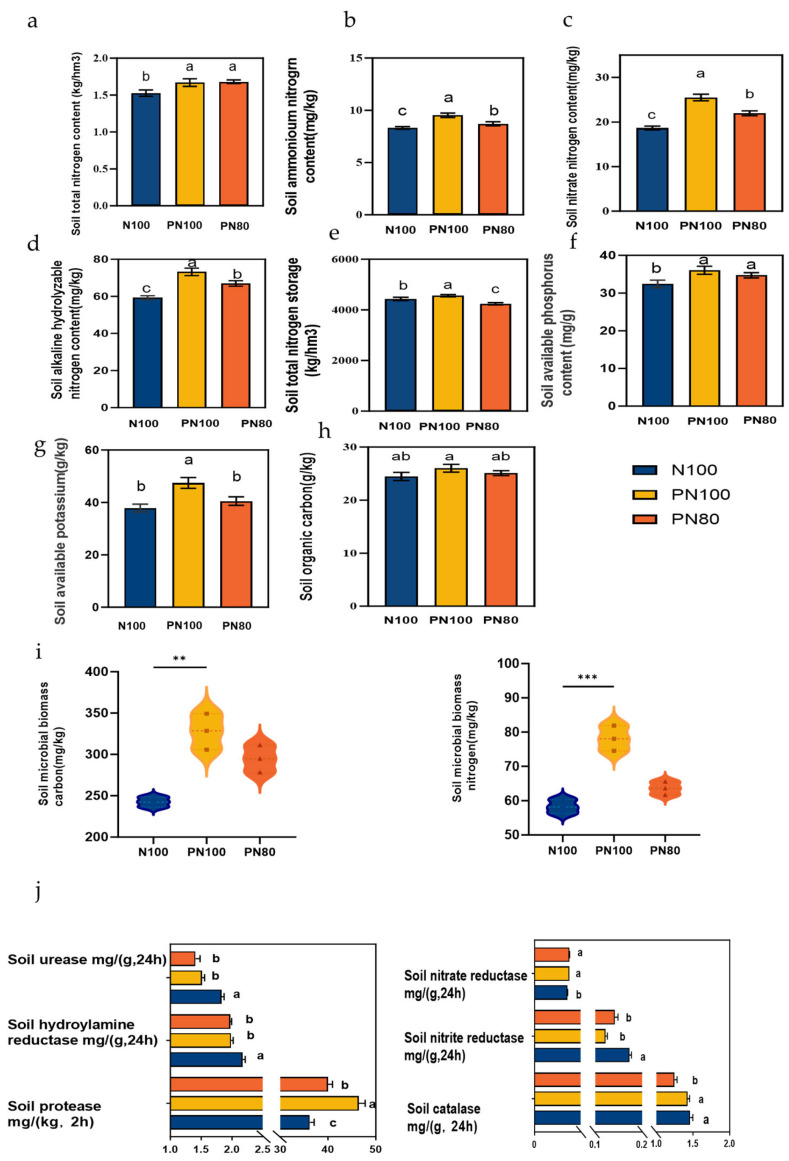
Changes in soil chemical properties and enzyme activities under different treatments: (**a**) Soil total nitrogen content; (**b**). Soil ammonium nitrogen content; (**c**) Soil nitrate nitrogen content; (**d**) Soil alkali hydrolyzable nitrogen content; (**e**) Soil total nitrogen storage; (**f**) Soil available potassium content; (**g**) Soil available phosphorus content; (**h**) Soil organic carbon content; (**i**) Soil microbial biomass carbon/nitrogen contents; (**j**) Soil enzyme activities. Different lowercase letters indicate significant difference between groups (*p* < 0.05). Asterisks indicates the level of significance (** *p* < 0.01, *** *p* < 0.001).

The soil TN, ammonium nitrogen, nitrate nitrogen, ANS, and AK contents of the PN100 treatment were 10.3%, 14.5%, 36.7%, 23.3%, and 21.7% higher than those of the N100 treatment, respectively (*p* < 0.05). The soil TN, ammonium nitrogen, nitrate nitrogen, ANS, and AK contents of the PN80 treatment were 9.03%, 4.71%, 17.8%, 12.8%, and 8.77% higher than those of the N100 treatment, respectively (*p* < 0.05). Moreover, the SOC, MBC, and MBN contents of the PN80 and PN100 treatments were higher than those of the N100 treatment, and the SOC, MBC, and MBN contents of the PN100 treatment were 6.36%, 35.2%, and 34.1% higher than those of the N100 treatment, respectively (*p* < 0.05) (Figure 3a–i).

The combined application of PPM and nitrogen fertilizer impacted the activities of soil enzymes. The nitrite reductase activity of the PN80 and PN100 treatments was 5.42% and 5.21% higher than that of the N100 treatment, respectively (*p* < 0.05), and the soil protease activity was 29.3% and 11.5% higher than that of the N100 treatment, respectively (*p* < 0.05). On the contrary, the activities of soil urease, hydroxylamine reductase, nitrate reductase, and catalase of the PN80 and PN100 treatments were lower than those of the N100 treatment (*p* < 0.05). Particularly, the soil catalase activity of the PN80 treatment was 15.1% lower than that of the N100 treatment (*p* < 0.05) (Figure 3j).

### 3.5. RDA of Soil Physicochemical Properties and Biocommunities

**Figure 4:**
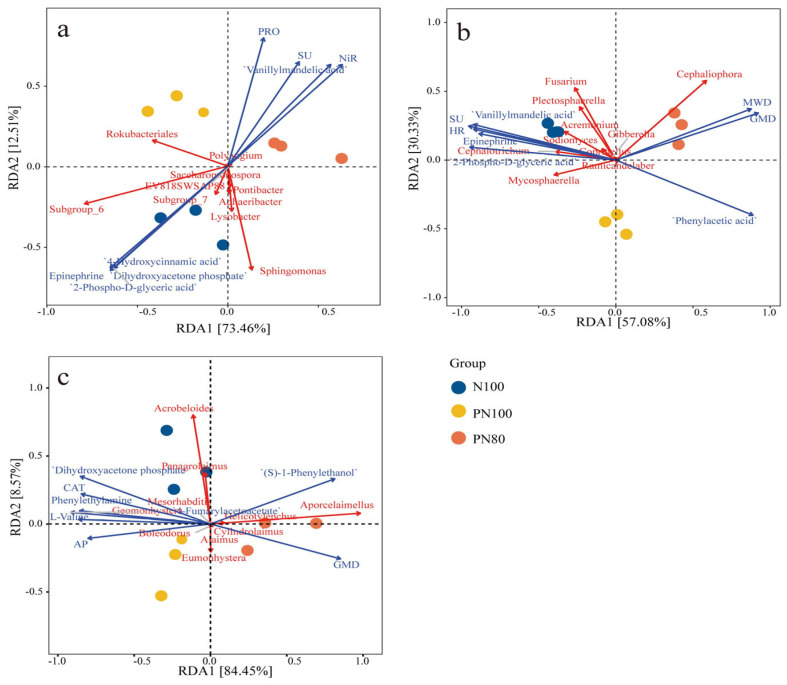
Redundancy analysis (RDA) of soil biocommunity and soil physicochemical properties: (**a**) RDA of soil bacteria and soil properties; (**b**) RDA of soil fungi and soil properties; (**c**) RDA of soil nematodes and soil properties.

RDA results showed that RDA1 and RDA2 axes explained 73.5% and 12.5% of the total variations in the bacterial community, respectively (Figure 4a). The RDA1 axis separated N100 and PN100 treatments from PN80 treatment, while the RDA2 axis separated N100 treatment from polymer treatments (PN100, PN80). Meanwhile, PN80 treatment had a significantly positive correlation with the activities of soil protease, urease, and nitrite reductase. The PN100 treatment mainly significantly impacted *Rokubacteriales.* Among soil environmental factors, the activities of soil protease (PRO), urease (SU), and nitrite reductase (NiR) had the greatest impact on the bacterial community structure. The abundances of bacteria *Rokubacteriales*, *Subgroup-6*, and *Sphingomonas* were significantly negatively correlated with the activity of PRO, SU, and NiR, while the abundance of *Rokubacteriales* was positively correlated with PRO activity.

RDA results showed that RDA1 and RDA2 axes explained 57.1% and 30.3% of the total variations in the fungal community, respectively (Figure 4b). The RDA1 axis separated N100 treatment from PN80 treatment, while the RDA2 axis separated PN100 treatment from N100 and PN80 treatments. Meanwhile, PN80 treatment mainly significantly impacted MWD, GMD, and *Cephaliophora* (*p* < 0.05) (Figure 4b). Among soil environmental factors, MWD, GMD, SU activity, and hydroxylamine reductase (HR) activity had the greatest impact on the fungal community structure. Soil MWD and GMD were significantly positively correlated with the abundance of fungus *Cephaliophora*, and negatively correlated with the abundance of fungus *Fusarium*. However, the abundance of *Fusarium* was positively correlated with soil SU and HR activities, and negatively correlated with soil MWD and GMD.

RDA results showed that RDA1 and RDA2 axes explained 84.5% and 8.57% of the total variations in the nematode community, respectively (Figure 4c). The RDA1 axis separated N100 and PN100 treatments from PN80 treatment, and the RDA2 axis separated N100 treatment from polymer treatments (PN100, PN80). The nematode community of the PN80 treatment was mainly affected by the abundance of predatory/omnivorous nematode *Aporcelaimellus* and the metabolite (S)-1-Phenylthanol. The nematode community of the PN100 treatment was mainly affected by the bacteriophagous nematode *Eumonhystera* and the available phosphorus content in the soil (Figure 4c). The soil environmental factors GMD, AP, and catalase activity had the greatest impact on the nematode community structure. The abundances of bacteriophagous nematodes *Acrobeloids* and *Panagrolainus* were positively correlated with soil AP content and catalase activity, but negatively correlated with soil GMD. The abundance of the predatory/omnivorous nematode *Aporcelainellus* was positively correlated with GMD and negatively correlated with soil AP content.

### 3.6. Soil Metabolome Characteristics and Pathway Analysis

**Figure 5:**
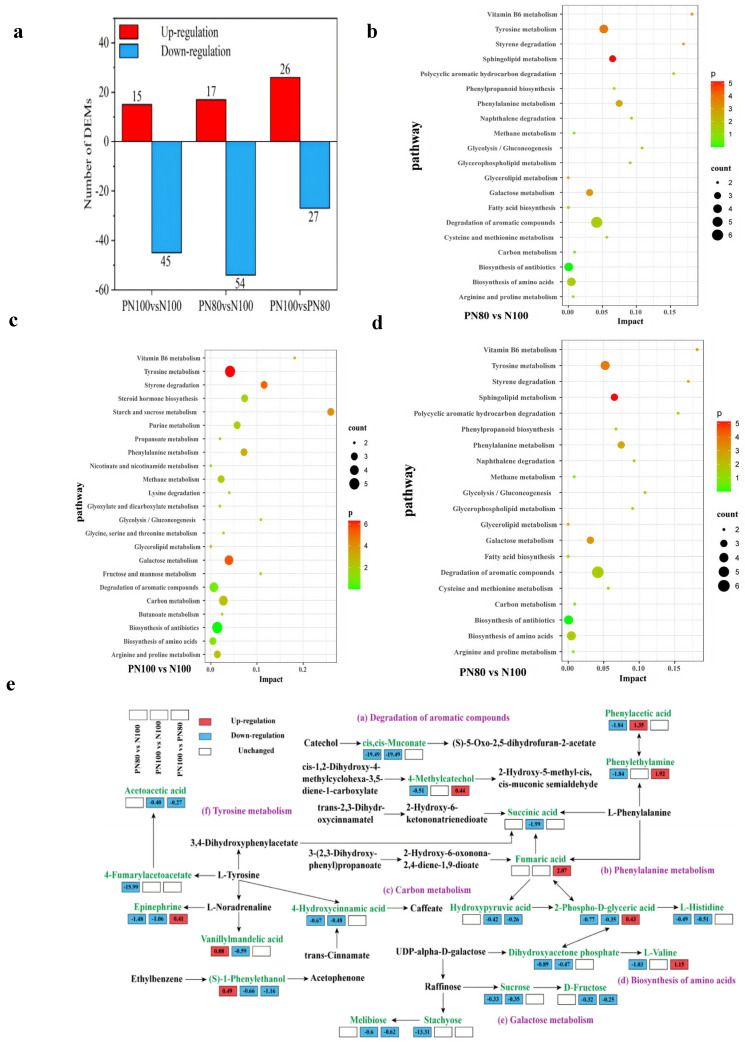
(**a**) Quantities of different metabolites in the soil under different treatments; (**b**) KEGG enrichment analysis of soil differentially abundant metabolites (DAMs) of PN80 vs. PN100; (**c**) KEGG enrichment analysis of DAMs of PN100 vs. N100; (**d**) KEGG enrichment analysis of DAMs of PN100 vs. PN80; (**e**) Analysis of soil metabolic pathways under different treatments.

There were 60 DAMs in PN100 vs. N100, among which 15 metabolites were up-regulated and 45 metabolites were down-regulated. There were 71 DAMs in PN80 vs. N100, among which 17 metabolites were up-regulated and 54 metabolites were down-regulated (Figure 5a). There were 53 DAMs in PN80 vs. PN100, among which 26 metabolites were up-regulated and 27 metabolites were down-regulated.

KEGG enrichment analysis (Figure 5b–d) showed that the DAMs for PN80 vs. N100 were significantly enriched in the Degradation of aromatic compounds, Tyrosine metabolism, Biosynthesis of amino acids, and Biosynthesis of antibiotics pathways (count > 3). The DAMs for PN100 vs. N100 were significantly enriched in the Tyrosine metabolism, Biosynthesis of antibiotics, Galactose metabolism, Carbon metabolism, and Degradation of aromatic compounds pathways (count > 3). The DAMs for PN100 vs. PN80 were significantly enriched in the Biosynthesis of antibiotics and Biosynthesis of secondary metabolites pathways (count > 3).

In the Degradation of aromatic compounds pathway (Figure 5e), the cis,cis-Muconate and 4-hydroxycinnamic acid metabolic pathways of the PN100 and PN80 treatments were significantly down-regulated compared with those of the N100 treatment, which slowed down the catabolism of aromatic compounds and led to the accumulation of intermediates. The 4-methylcatechol metabolic pathway of the PN80 treatment was significantly down-regulated, and the (S)-1-phenylethanol metabolic pathway was significantly up-regulated, compared with those of the N100 treatment. The succinic acid and (S)-1-phenylethanol metabolic pathways of the PN100 treatment were significantly down-regulated compared with those of the N100 treatment. In the Phenylalanine metabolism pathway, the phenylethylamine metabolic pathway of the PN80 treatment was significantly down-regulated, and the phenylacetic acid metabolic pathway of the PN100 and PN80 treatments was significantly up-regulated, compared with those of the N100 treatment. In the carbon metabolism pathway, the hydroxypyruvic acid metabolic pathway of the PN100 treatment was significantly down-regulated, and the 2-phospho-D-glyceric acid and dihydroxyacetone phosphate metabolic pathways of the PN100 and PN80 treatments were also significantly down-regulated, compared with those of the N100 treatment. In the Biosynthesis of amino acids pathway, the L-histidine and L-valine metabolic pathways of the PN100 and PN80 treatments were significantly down-regulated, compared with those of the N100 treatment. In the Tyrosine metabolism pathway, the epinephrine and vanillylmandelic acid metabolic pathways of the PN100 and PN80 treatments were significantly down-regulated, and the 4-fumarylacetoacetate metabolic pathway of the PN80 treatment and the Acetoacetic acid metabolic pathway of the PN100 treatment were also significantly down-regulated, compared with those of the N100 treatment.

### 3.7. Correlation Network Analysis

**Figure 6:**
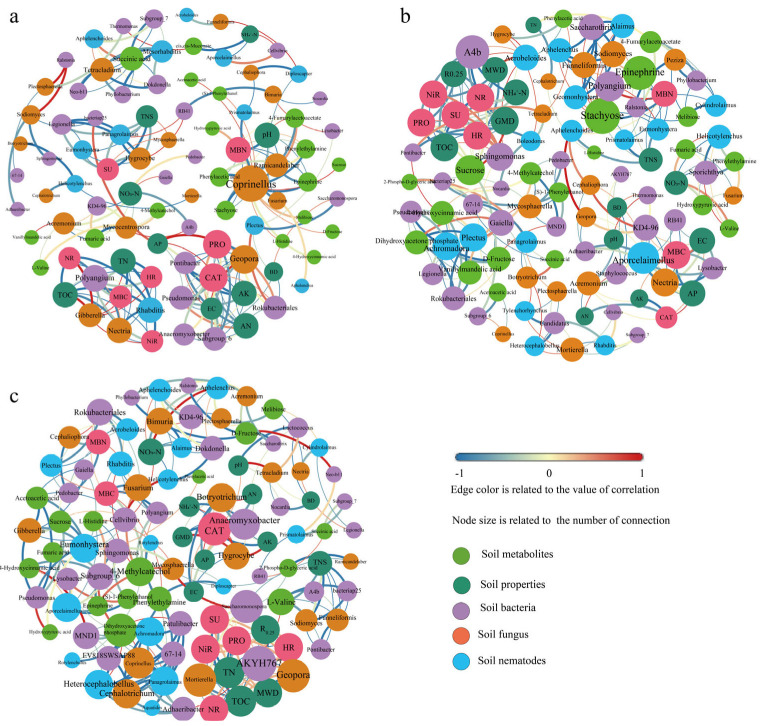
Network analysis of soil physicochemical properties, biological activities, and metabolites under different treatments: (**a**). N100 treatment; (**b**). PN100 treatment; (**c**). PN80 treatment.

Due to the complex responses of bacterial, fungal, and nematode communities for different treatments, the relationships of soil properties, key microorganisms, and nematode groups, and interactions between metabolites can be indicated by the symbiotic network analysis under different treatments (Figure 6). The PN100 and PN80 treatments had more nodes and edges in the network compared with the N100 treatment, indicating that the PN100 and PN80 treatments had a more complex network. Moreover, the network of the PN80 treatment was more complex than that of the PN100 treatment.

In the N100 treatment (Figure 6a), fungal communities including *Coprinellus*, *Ramicandelaber*, *Geopora*, *Gibberella*, and *Nectria* were network hubs. The N100 treatment had the lowest soil protease activity and the highest catalase activity among the treatments, due to the strong correlation between the two enzymes and soil physicochemical properties (AN, AP, AK, EC) and the interactions of AN, AP, AK, and EC with soil bacteria (*Pontibacter*, *Pseudomonas*, *Anaeromyxobacter*, *Subgroup_6*, *Rokubacteriales*) and fungi (*Geopora*). The abundance of the fungus *Coprinellus* (*Basidiomycota*) was negatively correlated with MBN, and the abundance of the fungus *Geopora* (*Ascomycota*) was positively correlated with soil AN content and catalase activity.

In the PN100 treatment, bacteria *(A4b*, *RB41*, *KD4-96*, *Lysobacter*, *Polyangium*), nematode (*Aporcelaimellus*), and metabolites (Stachyose, Epinephrine, Sucrose) were network hubs (Figure 6b). The soil TN, SOC, MBC, and MBN contents of the PN100 treatment were higher than those of the N100 treatment, and the activities of soil urease, hydroxylamine reductase, and nitrate reductase were lower than those of the N100 treatment, due to the metabolite Sucrose and the soil bacteria *A4b* and *Sphingomonas* (Figure 6b). The abundance of bacterium *A4b* (*Chloroflexi*) was negatively correlated with soil ammonium nitrogen and SOC contents. The abundance of bacterium *RB41* (*Acidobacteria*) was positively correlated with soil MBC and AP contents. The abundance of bacterium *Polyangium* (*Proteobacteria*) was negatively correlated with soil MBN and metabolites (Stachyose, Epinephrine).

Compared with N100 treatment, the PN80 treatment had the most significant change in microbial community, showing the most complex network. Bacteria (*AKYH767*, *Saccharomonospora*, *Anaeromyxobacter*, *MND1*, *EV818SWSAP88 67-14*), fungi (*Mortierella*, *Geopora*, *Cephalotrichum*, *Coprinellus*, *Hygrocybe*, *Botryotrichum*, *Fusarium*), and nematodes (*Achromadora*, *Heterocephalobellus*, *Rhabditis*, *Panagrolaimus*, *Plectus*) were network hubs (Figure 6c). The variations in the contents of TN and SOC of the PN80 treatment compared with the N100 treatment were not only related to soil bacteria (*AKYH767*, *Saccharomonospora*) and fungi (*Mortierella*), *Geopora*, but also to the activities of protease, urease, nitrate reductase, and hydroxylamine reductase. The abundances of bacteria *AKYH767* (*Bacteroidetes*) and *Saccharomonospora* (*Actinobacteria*) were positively correlated with soil TN and SOC contents, and the abundances of fungi *Mortierella* and *Geopora* were negatively correlated with soil TN and SOC contents (Figure 6c).

## 4. Discussion

In this study, PPM plays a significant role in regulating soil biocommunities, which are significantly influenced by nitrogen fertilization rates. PN100 treatment led to a significant reduction in the bacterial, fungal, and nematode diversity as well as the richness of bacterial communities compared with the N100 treatment. This result is consistent with the conclusion of Wang et al. [35] that high nitrogen fertilization rates reduce the soil bacterial abundance and diversity compared with the control. This indicates that nitrogen addition causes a reduction in soil bacterial abundance and diversity under PPM application. It is worth noting that PN80 treatment had higher bacterial and nematode diversity and richness compared with the N100 treatment. Due to polymers themselves having no adverse effects on soil microbial communities [36], PPM can have a positive impact on soil biocommunity structure under moderate nitrogen reduction conditions, maintaining soil biocommunity diversity. This finding is consistent with that of Tian et al. [14].

Soil microorganisms play an important role in driving processes such as soil carbon and nitrogen cycling, and organic matter formation and degradation [37]. The changes in soil microbial community structure can reflect the regulatory effects of soil environmental factors [38]. This study found that the application of PPM significantly altered the bacterial community structure; i.e., PN80 treatment promoted the enrichment of bacterial genera *AKYH767* (*Bacteroidetes*) and *Saccharomonospora* (*Actinobacteria*) and also led to a significant increase in the relative abundance of the beneficial bacterial genus *Sphingomonas* (*Proteobacteria*) compared with the N100 treatment. The phylum Proteobacteria can fix nitrogen, participate in organic matter mineralization [39], and promote plant growth [40]. *AKYH767* (*Bacteroidetes*) and *Saccharomonospora* (*Actinobacteria*) play an important role in the decomposition of soil organic matter, and their enrichment may accelerate the turnover of soil organic carbon [41,42]. Therefore, the increase in beneficial bacteria such as *Sphingomonas* may indirectly regulate carbon and nitrogen balance by improving the root zone microenvironment, promoting the accumulation of soil total nitrogen and organic carbon in the PN80 treatment in this study. Instead, *RB41* and *KD4-96* (*Chloroflexi*) were the main bacteria in the PN100 treatment. *Chloroflexi* plays an important role in organic degradation and carbon cycling processes [43]. The decrease in the relative abundance of *KD4-96* in this study may be due to the fact that PPM application may inhibit the metabolic activity of *KD4-96*, which reduces the rate of organic matter decomposition and leads to the accumulation of organic carbon [44]. This result is consistent with the conclusion of Holatko et al. [45] that organic amendments slow down carbon mineralization. This also further confirms the close relationship between soil properties and microbial communities.

The application of PPM significantly altered the fungal community structure and reduced the relative abundances of soil pathogenic fungi *Fusarium* and *Plectosphaerella* (*Ascomycota*) compared with the N100 treatment. Fungal communities are a key component of ecosystems, and their structural and diversity changes are directly related to soil-borne diseases and plant health [46]. Previous studies have shown that high soil fungal diversity is likely to lead to early root rot of wheat [47]. In addition, a study has shown [48] that the abundance of fungi *Ascomycota* in the rhizosphere soil of *Coptis chinensis* with root rot is significantly higher than that of healthy plants. *Fusarium* is a typical soil-borne pathogenic fungus. It is both a soil-borne pathogen and a facultative parasite, widely endangering crops [49,50]. The results of this study are consistent with the above research, indicating that the application of PPM may significantly inhibit pathogenic fungal communities in soil, reduce the soil-borne diseases, improve the root zone microenvironment, and promote plant health. However, the specific mechanism by which PPM inhibits the growth of pathogenic fungi still needs further investigation.

In addition, in this study, the application of PPM maintained a high diversity of soil nematode communities, especially in the PN80 treatment. Among the soil nematodes, the bacteriophagous nematode *Prismatoliaimus* actively responded to changes in the soil environment. *Prismatoliaimus* is a consumer that feeds on bacteria and plays an important role in organic matter decomposition and nitrogen mineralization [51,52]. Therefore, PPM may enhance the decomposition of soil organic matter and nitrogen mineralization and enrich the food web of soil nematode communities by regulating soil nematode community structure, enhancing soil nutrient cycling efficiency.

Soil metabolites, as important nitrogen sources for soil microorganisms, are closely related to the structure and metabolic pathways of soil biocommunities [53]. This study found that PPM combined with nitrogen fertilization significantly enhanced key metabolic pathways such as soil carbon metabolism and amino acid biosynthesis, and promoted the assimilation of nitrogen by microorganisms by up-regulating key metabolites Dihydroxyacetone phosphate and 2-Phospho-D-glycine acid in amino acid biosynthesis pathways. This further indicates that the application of PPM may maintain carbon and nitrogen balance by regulating soil biocommunity structure and metabolic processes [54,55]. In addition, this study found that there were differences in the nitrogen utilization strategies of soil biocommunities under the combined application of PPM and different nitrogen fertilization rates [56]. Specifically, N100 treatment significantly inhibited the activity of the Tyrosine metabolism pathway in soil, while PPM treatments significantly up-regulated the abundances of metabolites such as 4-Fumarylacetoacetate in this pathway. This indicates that the application of PPM can alleviate the inhibition of Tyrosine metabolism activity induced by nitrogen input, promoting soil carbon mineralization and enhancing nitrogen availability [57]. These results indicate that PPM regulates soil microbial communities and their metabolic pathways to enhance the expression of key metabolic pathways and the abundance of key functional metabolites, optimizing soil nutrient turnover and ecological functions.

## 5. Conclusions

The application of PPM significantly altered the diversity and composition of soil microbial, fungal, and nematode communities, and its regulatory effect was significantly influenced by nitrogen fertilization rates. Moderate nitrogen reduction (PN80) combined with PPM promoted the enrichment of beneficial biocommunities such as *Sphingomonas*, *Saccharomonospora* (*Actinobacteria*) and the predatory/omnivorous nematode *Aporcelaimellus*, enhanced the abundance of key metabolites related to soil carbon and nitrogen metabolism (Dihydroxyacetone phosphate, 2-Phospho-D-glycine acid), and optimized soil carbon and nitrogen cycling and organic matter turnover. Under the conventional nitrogen fertilization rate combined with PPM application (PN100 treatment), the activity of the key bacterial genus *KD4-96* (*Chloroflexi*) was significantly inhibited, leading to the accumulation of organic carbon. In addition, the application of PPM significantly reduced the abundances of pathogenic fungi *Fusarium* and *Plectosphaerella*. Thus, PPM may have potential value in the prevention and control of soil-borne diseases. The combination of PPM and nitrogen fertilizer significantly increased soil nutrient content (such as total nitrogen, alkali hydrolyzable nitrogen, ammonium nitrogen, nitrate nitrogen, available potassium, organic carbon, microbial biomass carbon, and nitrogen) and enzyme activity (protease, nitrite) by regulating soil microbial community structure, promoting the nitrification of soil nitrogen. Symbiotic network analysis further revealed that PPM enhanced the correlation between soil biocommunities, metabolites, and enzyme activities, regulated key functional pathways such as carbon metabolism, amino acid synthesis, and nucleotide metabolism, optimized the relationship between biocommunities and soil physicochemical properties, and helped enhance nitrogen-use efficiency and reduce nitrogen loss. Overall, nitrogen reduction combined with PPM application is a promising sustainable soil management strategy. However, the present study only explores the interaction between PPM and nitrogen fertilization rates, so future research should further explore the synergistic regulation of PPM and other nutrients such as phosphorus, potassium, and micronutrients. This will help promote the application of PPM in agroecosystems, stimulating sustainable agricultural development.

## Figures and Tables

**Table 1:** Experimental design.

Treatment	Description
N100	N fertilizer was applied at 300 kg·hm^2^ (traditional N application rate)
PN100	12 kg·hm^2^ of polymer PPM and 300 kg·hm^2^ of N fertilizer were applied
PN80	12 kg·hm^2^ of polymer PPM and 240 kg·hm^2^ of N fertilizer were applied

## Data Availability

The original contributions presented in this study are included in the article. Further inquiries can be directed to the corresponding authors.
